# Management Protocols for Spinal Injuries in Adult Patients: A Clinical Guideline: Management Recommendations for Patients with Spinal Cord Injuries

**DOI:** 10.31661/gmj.v14i.3783

**Published:** 2025-10-12

**Authors:** Esmael Amirazodi, Sharareh Jahangiri, Farshid Seraji, Ali Arianezhad, Zeynab Kord, Behrooz Zarasvand

**Affiliations:** ^1^ Department of Neurology, Ahvaz Jundishapur University of Medical Sciences, Ahvaz, Iran; ^2^ Department of Anesthesiology, Tehran University of Medical Science, Tehran, Iran; ^3^ Department of Neurology, Faculty of Medicine, Urmia University of Medical Sciences, Urmia, Iran; ^4^ Clinical Research Development Unit, Ganjavian Hospital, Dezful University of Medical Sciences, Dezful, Iran; ^5^ Research Center for Advanced Technologies in Cardiovascular Medicine, Cardiovascular Diseases Research Institute, Tehran University of Medical Sciences, Tehran, Iran; ^6^ Department of Nursing, School of Nursing , Dezful University of Medical Sciences, Dezful, Iran; ^7^ Department of Neurosurgery, School of Medicine, Dezful University of Medical Sciences, Dezful, Iran

**Keywords:** Spine Trauma, Spinal Cord Injury, Guideline, Adult Patients, Immobilization, Pain-Relieving

## Abstract

**Background:**

This research aimed to elucidate and establish management recommendations for patients with spinal cord injuries, tailored to the characteristics of Iranian society, through an exploratory mixed-methods study.

**Materials and Methods:**

The present study was executed in three phases. The initial phase was conducted to identify and elucidate the principal recommendations for the management of spinal cord injuries, comprising two sub-phases: a systematic review and a qualitative guided study. In the second stage, management recommendations were developed by integrating the findings from the first stage, and in the third stage, the clinical guide was prioritized and validated through two rounds of the Delphi method.

**Results:**

During the initial phase of the study (qualitative-review phase), 781 codes were derived from interviews with specialists in emergency medicine and neurosurgery, which were subsequently consolidated into six categories. During the second phase of the study, which involved the preparation of the primary draft, a review of pertinent texts was conducted, and researchers assessed 23 chosen clinical guidelines. In the third phase of the study (combination), the findings from the preceding two phases were integrated, and the questionnaire developed by 27 relevant experts was evaluated, culminating in the creation of the final clinical guide.

**Conclusion:**

This clinical guide, encompassing six categories—Pre-hospital, Immobilization, Diagnostic Imaging, Pain Management, Medication Recommendations, and Surgical Intervention—has been developed and compiled for the utilization of physicians in the emergency departments of hospitals in Iran.

## Introduction

Trauma accounts for over five million deaths worldwide each year and is the primary cause of death and one of the main causes of disability in developing nations [1][2]. By 2030, trauma-related mortality is also expected to rise by 40%, according to the World Health Organization [3]. Among the various forms of trauma, central nervous system trauma is the most common cause of hospitalization [4]. Spine injuries include both bone injuries and spinal cord injuries. Spinal cord injury is linked to spinal cord contusion, which occurs after a traumatic event as a result of a vertebral column fracture or dislocation [5]. In the United States, it is estimated that 2-6% of trauma patients have spinal cord injuries, and approximately one-third of these patients also have an unstable spinal cord fracture or spinal cord injury [6].

Furthermore, roughly 23% of all spine injuries are spinal cord injuries [7]. 1.6% of patients with spinal fractures had traumatic spinal cord injuries, according to a 2020 Iranian study [8]. Despite being uncommon when compared to the overall statistics of trauma-related injuries, spine injuries can result in high mortality rates as well as physical, social, and financial difficulties over the course of a person's lifetime [9]. Traumatic spinal cord injuries are divided into primary and secondary phases in order to improve services [10]. Compression of the spinal cord, stretching of nerve tissues, or disturbance of the local blood supply are the main causes of injuries. Primary injuries are brief and indicate direct harm to endothelial cells, supporting tissue, or neurons [11]. Ischemia, tissue inflammation, and excitotoxicity are secondary injuries that eventually result from primary injuries [12][13][14]. In order to benefit from trustworthy scientific evidence, physicians must follow a variety of clinical guidelines when it comes to prevention, diagnosis, treatment, and care [15]. Following a clinical guideline step-by-step is necessary for improved spinal cord injury treatment. Clinical guidelines developed in particular organizational, racial, and cultural contexts are used and systematically modified for use in other contexts [16]. It will be helpful to conduct research in this field that considers all human, social, and cultural factors and concerns [17].

This study aimed to collect and localize targeted recommendations for clinicians to manage spinal injuries in patients, grounded in credible scientific evidence. Despite the existence of numerous global guidelines on this issue, we are unable to identify a pertinent guideline applicable to the Iranian context and patients experiencing this trauma. Consequently, we resolved to conduct the present study utilizing a "outcome-based research" methodology to elucidate and establish therapeutic recommendations for patients with spinal cord injuries.

## Materials and Methods

**Figure-1:**
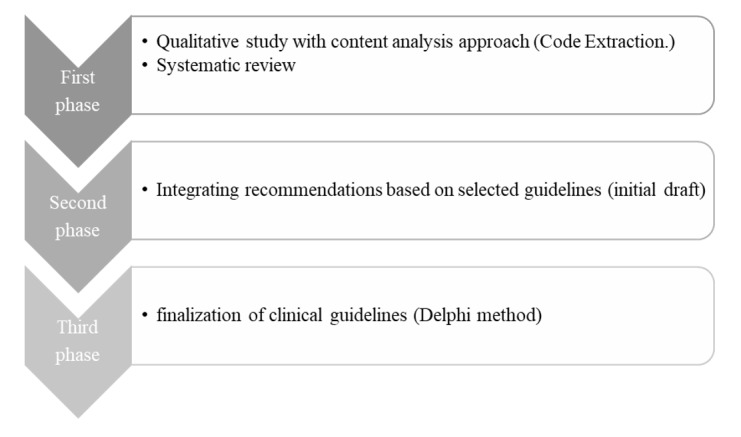
Figure 1. exploratory mixed-method

The present study is a type of health system research (HSR) that was designed and conducted using a mixed exploratory method in three stages from December 22nd, 2019 to July 9th, 2022 [18]. The study steps are shown in Figure-1.

### First Phase

The study's initial phase consisted of two smaller investigations. The first sub-study sought to "explain the perception of stakeholders (doctors) on management evidence for patients with spinal cord injuries." It used a contractual content analysis approach. Inclusion criteria for Participants who were chosen from among emergency medicine specialists and neurosurgery specialists who met the following criteria: expertise and experience in the field of care, diagnosis, prevention, and treatment of patients with spinal cord injury; two years or more of clinical work experience; willingness to cooperate in conducting research; and availability of time to cooperate in conducting research. Participants were chosen based on the research community's purpose-based sampling method. Elo Kingas' method was used to analyze the data in three stages: preparation, organization, and reporting. The criteria outlined by Lincoln and Goba, namely acceptability (prolonged engagement with the data, spending enough time, combining several methods (interview, observation), review and review by the Peer Check research team), reliability (use of The ability to be audited by an external observer from the stages of conducting the research, verifiability (auditing the research by the audience and readers in detail), and transparancy (auditing the research by the audience and readers in detail), were used to achieve the [19]. The interviews was done by authors that the important questions include: What do you think of SCI disease, What do you have experiences about treatment and diagnose of this patients, What are the prominent and serios problems SCI patients, Do you have any recommendations to improve the condition of this patients, What is your opinion about others recommendations which are suitable to Iranian patients missing in universal guidelines.

The second sub-study was conducted with the aim of "reviewing clinical guidelines and related protocols". Search using the keywords "Spinal trauma", "Spinal injury", "Adult patients", "Guideline", "traumatic spinal injury", "Practical "guideline" protocol", "pathway", "Care plane", "recommendation", "procedure", "standard", "clinical practice guideline" was searched (Table-1) between 2010-2022 in databases (Scopus, PubMed, Web of sciences, ProQuest, CINAHL, Medline Plus, EMBASE, Cochrane Library, Google Scholar), important specialized journals in the field of brain trauma and spinal cord injuries, scientific and administrative documents, reports and the World Health Organization website, government websites, and other authoritative websites, important national websites and an area related to organizations active in the field of spinal cord injuries were investigated. To be more precise, they include relevance to management of patients with SCI, accessible full-text, and publication in English or Persian between 2010 and 2022.

The quality of the retrieved guidelines will be assessed using the Appraisal of Guidelines for Research and Evaluation (AGREE) I instrument. Identification and validation of clinical guidelines and related protocols were done in three steps: formulating clinical questions and searching for clinical guidelines, evaluating clinical guidelines extracted by the AGREE-1 tool, and extracting recommendations. This tool examines accuracy and transparency in the content and structure of clinical guidelines. The purpose of using this tool is to evaluate, critique, and provide a framework for examining the quality of clinical guidelines, which includes 23 main items in six areas, including vision and purpose, stakeholder participation, accuracy and methodological quality, expressiveness and presentation, applicability, and independence in It is editing [20].

### Second Phase

The results of the study's initial phase, which involved interviews with stakeholders and a review of manuals and associated literature, were now being combined. The recommended conclusions were created as a six-section questionnaire based on clinical guidelines, guidelines, participant comments, and World Health Organization guidelines. Then, a list of management suggestions for patients with spinal cord injuries was created and distributed to the experts for Delphi method prioritization.

### Third Phase

This step was carried out in two rounds to "prioritize management recommendations for patients with spinal cord injuries" in the form of a classic Delphi panel using the ten steps proposed by Fowl [21]. The questionnaire prepared in the second phase for the first round of Delphi was given to 27 experts, experts, and policymakers who had enough experience and knowledge to comment on the topic under discussion and expressed their willingness to participate in the study. The members of the Delphi panel in this research were selected based on the purpose and using the snowball sampling method. They were asked to rank the indicators in the questionnaire according to their importance based on their views and experience; prioritize very high (4), high (3), medium (2), and low (1).

At this stage, it was analyzed using SPSS software and descriptive statistics, and the average scores of each index were determined. Therefore, based on the approach of similar studies [22][23]. Indicators with a high average (3 and above) were extracted. In the second round of Delphi, the priorities identified in the first round were examined to determine the importance, scientific and operational acceptability of care dimensions. Priority dimensions in the form of a questionnaire were provided to the panelists of the first round of Delphi through e-mail.

The participants were asked to express their opinions about each of the indicators and for each criterion separately and in a range including low (1), medium (2), and high (3). Also, at this stage, descriptive statistics and SPSS software were used for data analysis. Finally, after gathering the opinions of the expert group, agreeing or disagreeing with each of the options by the mean and standard deviation, the options that had an average of less than 2 were removed. Finally, the options that were agreed upon by the panel group were used as management recommendations for patients with spinal cord injuries in the form of guidelines.

### Funding

This article is a part of a thesis of the corresponding auther ZEYNAB KORD ,which was financically supported by Dezful university of medical sciences MED-400014-1400 .in this article we have received funding from vice chancellor of research from DEZFUL university of medical sciences.

## Results

**Chart-1:**
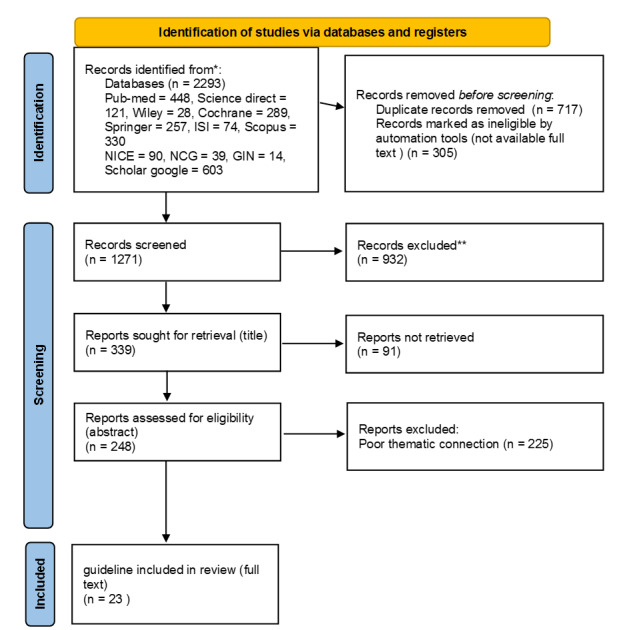
Chart 1. Prisma flow-chart depicting the literature search and selection of included and excluded studies

**Table:** Table1. Management Recommendations for Patients with Spinal Cord Injury

Pre-hospital and ABCDE
**1. Collection of vital information ** **2. Inform the trauma team ** **3. Granting responsibility and dividing tasks to each team member by the trauma team leader ** **4. Declaration of readiness to accept a trauma patient ** **5. Ensuring the existence and use of personal protective equipment (PPE) ** ABCDE protocol · **Protect the airway ** · **Assess aspiration risk ** · **Prevent bradycardia ** · **Protect the spine during the procedure and carry out diagnostic and therapeutic measures by maintaining the stability of the spine. ** · **Maintaining SpO2 between 94% and 98% ** · **Fixing two peripheral catheters. If necessary, fluid therapy and blood transfusion should be done. ** · **The patient's GCS should be evaluated and recorded, and changes in the GCS level should be noted. ** · **Physical examination and environmental factors control **
Cervical immobilization and necessity for radiological evaluation (Canadian C-spine rule)
**1. ** **GCS<15 during the emergency evaluation** **2. ** **Paralysis, peripheral focal nerve damage, or paraesthesia in the limbs ** **3. ** **Patients with neck pain and any of the risk factors include ** **:** - **Age ≥ 65 years** - **Falling from a height of more than one meter or 5 steps** - **Applying axial pressure to the head: for example, diving ** - **High-speed vehicle accidents (speed over 96-100 Km/h)** - **Overturned vehicle** - **Thrown out of vehicles** - **Accidents with recreational vehicles, or bicycle accident ** - **There is a vertebral disease, such as ankylosing spondylitis, rheumatoid arthritis, spinal canal stenosis, and previous neck surgery. ** **4. ** **Patients with a dangerous mechanism of injury (above) and a visible injury above the clavicle or a severe painful chest injury with a magnitude greater than 7 out of 10 in the absence of neck pain or tenderness. ** **5. ** **Severe neck pain with intensity greater than 7 out of 10 **
Immobilization in injury to the thoracic or lumbosacral vertebrae
· **In addition to the cases mentioned in the case of neck injury, immobilization of the spine is also required in the following cases. ** **1. ** **There is a risk of osteoporosis or a pathological point in the patient that makes the patient susceptible to bone fracture, for example, the use of corticosteroids. ** **2. ** **It is suspected that the spine is fractured in another area of the spine. ** **3. ** **It has abnormal symptoms (weakness or numbness). ** **4. ** **The presence of danger signs in the examination: ** - **Abnormal neurological symptoms (motor or sensory deficits). ** - **New shape change or touch treatment in the interosseous line of the spine. ** - **The middle bony index of the spine (in tapping). ** - **Pain in the midline or spine (when coughing). **
Immobility during intubation
1. **The existence of any injury that causes a disturbance of the senses ** **.** **2. ** **The patient is under the influence of drugs or alcohol ** **.** **3. ** **The patient does not react to external stimuli, is confused, or does not cooperate ** **.** **4. ** **The patient's level of consciousness has decreased ** **.** **5. ** **There is any pain in the spine** **.** **6. ** **Presence of any weakness in hands and feet (motor evaluation) ** **.** **7. ** **Mentions sensation of change or absence of sensation in hand or foot (sensory assessment) ** **.** **8. ** **Existence of priapism** **.** **9. ** **Has a history of previous spinal problems, including previous spinal surgery or conditions that predispose to spinal instability ** **.** · **If there are any of the mentioned cases, or if it is not possible to perform these evaluations, complete linear immobilization of the spinal cord must be performed. **
**Imaging**
**CT scan or X-ray**
· In children (under 16 years old), it is better to do simple radiography along with X-rays. · For adults (16 years and older), a CT scan is recommended in the following cases: 1. imaging of the cervical spine injury had been proven according to the criteria of the Canadian C-Spine Act. 2. Strong suspicion of thoracic or lumbosacral spine injury based on abnormal neurological signs or symptoms. 3. Suspected injury to the chest or lumbar spine (children and adults) 4. If the X-ray imaging is abnormal or there were clinical signs of spinal cord injury, a CT scan should be performed. 5. If a new vertebral fracture is confirmed, the rest of the spine should be imaged. · For suspected spinal cord injury without symptoms or abnormal neurologic signs in the thoracic or lumbosacral region (T1-L3), plain radiography is the first line of investigation.
**Whole body CT scan**
· Whole body CT scan (consisting of head-to-toe scanogram and then head-to-mid-thigh CT) is best used in adults (16 years or older) with blunt trauma and suspected multiple injuries. Patients should not move during whole-body CT scan. · It is recommended to use clinical findings and sonogram to guide limb CT in adults (16 years or older) with limb trauma. · Generally, whole-body CT should not be used for imaging children (under 16 years old). However, it is best to use clinical judgment to limit CT to the areas of the body where evaluation is needed.
**MRI**
· In the following cases, for children (under 16 years old), the diagnostic MRI modality was used: 1. Cervical spinal cord injury. 2. Damage to the cervical spine as shown in the clinical evaluation of abnormal neurological symptoms. 3. Consult a radiologist about plain X-ray findings and need more imaging. · In adults, if there is a neurological abnormality, regardless of whether the cause of this deficit is evident on CT or not, MRI can be used after a CT scan.

**Table:** Table2. EMS; Emergency Medicine Specialist, ABCDE; Airway, Breathing, Circulation, Disability, and Exposure

		EMS (n=11)	Neurosurgeon (n=16)	Total (n=27)
**Number**		11	16	27
**Clinical experience (mean/years)**		7.27 years (Rang; 5 to 10.5 years)	7.56 (Rang; 5.5 to 11 years)	7.41 (Rang; 5 to 11 years)
		Delphi method		
**Recommendations 1** **(Pre-hospital and ABCDE)**	Reliability	3.90	3.93	3.92
	Importance	3.9	3.93	3.92
	Application	3	3.26	3.13
	Facility	2.63	3	2.81
**Recommendations 2** **(Immobilization)**	Reliability	4	4	4
	Importance	4	4	4
	Application	4	4	4
	Facility	3.18	3.4	3.29
**Recommendations 3** **(Diagnostic imaging)**	Reliability	3.36	3.33	3.34
	Importance	3.63	3.53	3.58
	Application	3.09	3.13	3.11
	Facility	3.54	3.46	3.50
**Recommendations 4** **(Pain-relieving)**	Reliability	2.54	2.4	2.47
	Importance	2.90	2.86	2.88
	Application	2.63	2.66	2.65
	Facility	3.00	2.93	2.96
**Recommendations 5** **(Medication recommendations)**	Reliability	3.27	2.13	2.70
	Importance	3.27	2.12	2.69
	Application	3.36	2.36	2.86
	Facility	3.45	3.41	3.43
**Recommendations 6** **(Surgical intervention)**	Reliability	3.36	3.40	3.38
	Importance	3.27	3.33	3.30
	Application	3.00	3.13	3.06
	Facility	3.09	2.93	3.01

### Results of the First and Second Phase

In the first sub-study, 781 codes were extracted from the analysis of the data obtained from the interviews, and by removing duplicate codes and merging similar codes, 6 classes and 23 sub-classes were obtained. The relevant recommendations are summarized in Table-1.

In the second study, to extract the appropriate clinical guide, first the clinical question was designed based on the method (PIPOH), and 23 clinical guides, Clinical Practice, and evidence-based programs related to the purpose of the study were extracted with the relevant keywords (Chart-1). The retrieved clinical guidelines were initially evaluated by the researchers, and the quality of 23 guidelines was evaluated using the Persian version of the critical evaluation tool and AGREE research guide (Table-2). After selecting the evaluated clinical guidelines and reviewing the sources and evidence, relevant recommendations of 361 indicators were extracted.

### Results of the Third Phase

In this section, 361 indicators were created from the extracted indicators linked to management recommendations for patients with spinal cord injuries, which included 23 components (subcategories). This phase saw the completion of 27 surveys. There were still 361 indicators because none of the indicators had an average that was less than 3. The second round of Delphi validation examined the management axes for patients with spinal cord injuries in terms of relevance level, scientific acceptability, and operational acceptability. The total number of indicators stayed at 361 because none of the indicators had an average below 2. The matching average of the three criteria for each section's importance, scientific acceptability, and implementation capacity is shown in Table-2.

## Discussion

This clinical guide on six categories; Pre-hospital and ABCDE, Immobilization, Diagnostic imaging, Pain-relieving, Medication recommendations, and Surgical intervention were finalized.

### Pre-hospital and ABCDE

Patients with acute illnesses should undergo assessment and treatment utilizing a systematic approach grounded in ABCDE evaluation, as indicated by prior research. The objective of this evaluation for a SCI patient with possible concomitant trauma-related complications is to identify any potentially life-threatening conditions [24][25][26][27][28].

### Immobilisation

Research on immobilization has shown that secondary injuries resulting from improper patient transfer account for over 25% of spinal cord injuries [29]. In light of this, hospital treatment of these injuries can play a critical role in reducing the trauma-induced mortality and morbidity [30]. The available documents were considered insufficient for consensus, unlike some immobilization studies [31]. Our study's findings are consistent with the systematic review by Christian et al., which concluded that clinical findings about the mechanism of the injury are more significant when deciding whether to immobilize [32]. Additionally, maintaining immobility throughout treatment requires clinical discretion. Prior to surgery, it should evaluate the patient's risk of harm, considering the possibility of spinal cord injury from the intubation procedure [33]. See Table-1.

### Medication Recommendations

According to the experts in this study, there is an inverse relationship between the prognosis of people with spinal cord injuries and cardiovascular problems. Specifically, sympathetic nervous system disruption, which usually occurs in individuals with severe spinal cord injury at T6 or higher, can cause hypotension and cardiac arrhythmias (often bradycardia) [34]. In this population, vasopressors and the use of crystalloid fluids can be used to increase blood pressure [34]. Laboratory data indicates that hypotension leads to insufficient spinal cord perfusion, which exacerbates secondary damage and worsens neurologic outcomes [35], ultimately compromising patient outcomes [36]. Insufficient randomized trials were performed to evaluate the neurological outcomes of patients with spinal cord injuries at a specific blood pressure target [37]. Previous research on the treatment of acute traumatic spinal cord injury (SCI) recommends artificially elevating the patient's mean arterial pressure (MAP) to exceed 85 mm Hg for a duration of seven days to enhance blood flow to the injured spinal cord. Norepinephrine is a viable alternative for this purpose [38][39][40][41]. Additional clinical trial research is necessary in this domain due to the insufficient high-level evidence to derive more precise conclusions.

The administration of high-dose intravenous methylprednisolone sodium succinate (MPSS) during the acute phase of injury has traditionally been the most controversial issue concerning the medical treatment of spinal cord injury (SCI). MPSS has been shown to act as a neuroprotective agent in preclinical studies [42][43][44]. Although methylprednisolone offers preventive advantages for spinal cord injury (SCI) patients, further research revealed no significant difference in recovery between those receiving methylprednisolone injections and those who did not [45]. Consequently, the use of methylprednisolone, nimodipine, or naloxone for neuroprotection or the prevention of secondary degeneration after traumatic spinal cord injury is not recommended [41][46][47]. Evidence indicates that administering methylprednisolone to adult patients within eight hours of acute spinal cord injury is a feasible therapeutic option [48][49]. Participants in the current project did not validate the efficacy of glucocorticosteroid injection.

### Diagnostic Imaging

For patients with spinal injuries, choosing the most effective diagnostic method is essential. MRI is considered the gold standard for detecting spinal cord injuries in comparison to radiography, CT scans, and other diagnostic methods, although it is not always the primary choice due to its limitations [50][51]. Numerous studies indicate that MRI is more beneficial than CT scans in assisting surgeons' assessments and in identifying spinal cord and soft tissue injuries in patients with spinal cord injuries [52][53][54]. Consult Table-1, which is based on the perspectives of emergency medicine specialists and neurosurgeons, to make a decision regarding this field.

### Surgical Intervention

The choice of surgical intervention is an additional subject addressed in the clinical handbook. The fundamental cause, treatment responsiveness, and timing of therapeutic intervention all influence the patients' prognosis. Previous research indicates that 70% of individuals with traumatic lumbosacral plexus injury achieve spontaneous recovery within 18 months [55]. Nonetheless, early surgical intervention is favored [56], Surgical decompression conducted within 24 hours of an acute spinal cord injury is associated with sensory-motor recovery [57]. The initial 24-36 hours following acute spinal cord injury, subsequent to decompression surgery, appear to be a critical period for optimal neurological recovery [57]. A meta-analysis of 16 trials indicates that acute SCI patients experience superior recovery following early surgery compared to late surgery, resulting in enhanced neurological recovery, reduced hospital duration, and fewer complications [58]. The findings of another meta-analysis not only endorsed early surgery but also emphasized the necessity for further research in this domain [59]. Emergency surgical decompression should be performed within 24 hours of the onset of neurological deficits in patients with traumatic spinal cord injuries or lumbosacral plexus injuries, as per the available resources. The challenge in implementing this advice arises from an absence of suitable tools, proficient personnel, and competent surgeons to perform the necessary procedure..

### Pain-relieving

Recent research indicates that individuals with spinal cord injuries infrequently receive pain medication, potentially infringing upon their legal rights. Nonetheless, profound sedation may lead to organ dysfunction, disorientation, and respiratory depression. Treating neuropathic pain is more complex than addressing musculoskeletal pain [60]. In the absence of identifying the primary cause of the pain, neuropathic pain is predominantly managed symptomatically [61]. Furthermore, the study indicates that approximately 70% of spinal cord injury patients endure chronic discomfort [60]. Non-steroidal anti-inflammatory drugs (NSAIDs) are administered for acute pain based on extensive clinical experience [62]. Take into account the side effects in this regard.

## Conclusion

**Figure-2:**
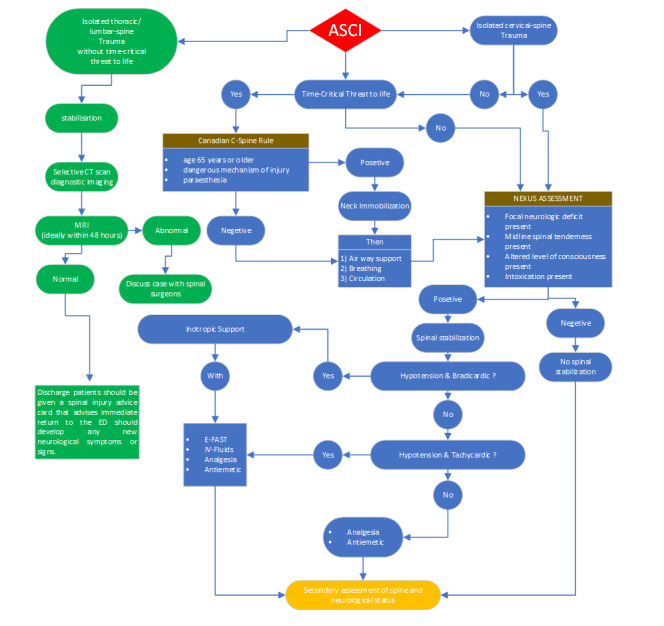
Figure 2. primary assessment of spinal cord injury patients

**Figure-3:**
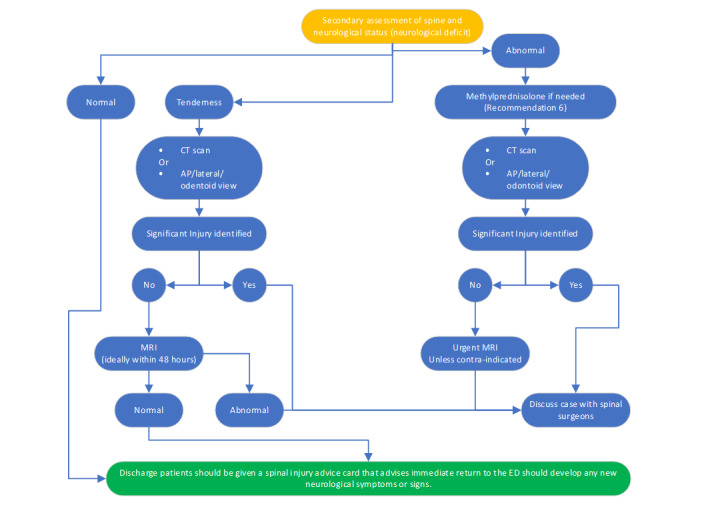
Figure 3. secondary assessment of spinal cord injury patients

One of the most formidable and critical obstacles in the care of adult patients is the management of spinal injuries, which have dire long-term consequences for both the patient and society [63]. The accurate identification and management of these injuries has become increasingly vital due to the increase in adult life expectancy. Adult spinal cord injuries have undergone extensive research; however, certain facets of their management remain ambiguous [63]. Secondary injuries can be particularly mitigated through immediate and effective resuscitation, which reduces tissue hypoxia and blood pressure [63][64][65]. Long-term patient outcomes can be enhanced through a multidisciplinary approach that integrates all relevant specializations in the treatment of traumatic spinal cord injury [64].

This clinical guideline facilitates the comprehensive and effective management of spine injuries in adult patients. This recommendation was formulated based on credible data and scientific research, considering the distinctive characteristics of this age group. This guideline addresses the management of spinal cord injuries in adults through various modalities, encompassing clinical assessment, immobilization, diagnostic imaging, selection of nonsurgical and surgical interventions, and pain alleviation. (Figures -2 and -3).

Following the evaluation of the initial category of evidence, an effort was made to consolidate optimal care-treatment recommendations utilizing the latest studies, which were subsequently modified in consultation with relevant experts to align with the available facilities in Iran. This was executed to enhance the management quality of spine injury patients and diminish the costs linked to conventional spinal injury management.

The primary objectives of this recommendation are to improve clinical outcomes, reduce side effects, and expedite the recovery process for adult spinal cord injury patients. This recommendation serves as a practical reference for medical professionals, including physicians, orthopedic surgeons, physiotherapists, and other treatment teams, when managing adult patients with spinal cord injuries. Through the issuance of this clinical recommendation, we aim to.

## Conflict of Interest

No competing interests are declared by the authors.
